# The dynamics of electron beam scattering on metal membranes: nanopore formation in metal membranes using transmission electron microscopy

**DOI:** 10.1186/s40580-018-0164-z

**Published:** 2018-11-12

**Authors:** Hyun-Mi Kim, Kyeong-Beom Park, Hyung-Jun Kim, Hongsik Chae, Jae-Seok Yu, Kidan Lee, Ki-Bum Kim

**Affiliations:** 1 Research Institute of Advanced Materials (RIAM), Seoul National University, Seoul, South Korea; 2 Department of Materials Science and Engineering, Seoul National University, Seoul, South Korea

**Keywords:** Nanopore perforation, Focused e-beam, Scattering cross-section

## Abstract

The dynamics of nanopore formation in metal membranes using the highly focused and high energy electron beams (e-beams) of transmission electron microscopy instruments was investigated. Various metals such as Al, Ti, Cr, Cu, and Au were selected to investigate the effect of the atomic mass of the metal on nanopore drilling, namely, elastic versus inelastic scattering. We demonstrated that the effect of elastic scattering (pore formation by sputtering) decreased as the atomic mass of the metal increased. Furthermore, experimental cross-sections obtained from normalized drilled volume vs. electron dose curves (characteristic contrast curves) matched well the calculated atomic displacement cross-sections determined from elastic scattering data. The sputtering energies of Ti, Cr, and Cu were determined to be approximately 10, 9, and 7 eV, respectively, which were in good agreement with the reported range of sputtering energy values.

## Introduction

Experimentally validating the idea of reading the sequence of nucleic acids of a single strand DNA by detecting the associated ionic current drop while the DNA is electrically driven through a nanopore is conditioned by the successful forming of nanometer scale pores in thin membranes. The work of Dekker et al. [1] who first reported the successful formation of nanopores in SiN_x_ membranes using highly focused electron beams (e-beams) during transmission electron microscopy (TEM) studies, led to nanopores becoming one of the mainstream technologies for detecting small biomolecules in aqueous solutions. Prior to their study, there have been several reports about direct nanoscale sculpturing using the focused e-beams in TEM instruments [2]. In particular, material changes, typically referred to as “damage” caused by the high energy e-beams during TEM analysis have been a concern researchers have tried to understand and resolve for a long time [3, 4]. Efforts have been made to explain the damage employing the concept of energy transfer from the incident high energy particle (electron) to the rest of the atom. The perforating (sputtering) phenomena caused by the focused e-beam was also explained as a type of “damage” to the very thin material [5].

There are two different types of energy transfer processes the e-beam in TEM can be involved in: radiolysis due to the inelastic scattering of the electron–electron interactions resulting in specimen heating, ionization, and X-rays or Auger electrons generation, and knock-on damage due to the elastic scattering of the electron-atom interactions resulting in atomic displacement creating point defects within the materials or sputtered atoms at the surface of the specimens. Generally, it is known that the degree of energy transfer by radiolysis is reduced at higher e-beam energies while that of the knock-on damage is increased [3]. In our previous report [5], we summarized the theoretical prediction of each process (ionization, heating, and direct atomic displacement) on the energy transfer from high energy electrons to target materials based on the scattering cross-section, i.e., scattering probability, direct atomic displacement cross-section ($\upsigma_{\text{d}}$) due to elastic scattering considering the relativistic kinematics of binary collisions (the Mott formula), and inelastic scattering cross-section ($\upsigma_{\text{in}}$) using the continuous slow down approximation (the Bethe formula). Here, $\upsigma_{\text{d}}$ is a function of the incident e-beam energy (E), atomic mass (M) of the target membrane and displacement energy (E_d_), which is the kinetic energy required to displace an atom from its original lattice position to an unstable one. Moreover, $\upsigma_{\text{in}}$ is a function of E, M, and the electron excitation potential (I). From calculating $\upsigma_{\text{d}}$ as a function of E, it was concluded that $\upsigma_{\text{d}}$ started to increase at the critical e-beam energy and continued to increase with increasing the electron beam energy, while $\upsigma_{\text{in}}$ decreased with the increase in electron beam energy. We theoretically calculated $\upsigma_{\text{d}}$ and $\upsigma_{\text{in}}$ for SiN_x_ membranes and experimentally demonstrated that nanopore drilling in SiN_x_ membranes by the incident electron beam energy was the result of direct atomic displacement. The direct atomic displacement energy of SiN_x_ (17 eV) could be determined from the evolution of nanopores as function of the energy and dose of the e-beam, and was well matched with the sputtering energy.

Solid-state nanopores using SiN_x_ membranes are extensively adopted because of their simple fabrication process on Si substrates and easy perforation using TEM. However, the interest for metal embedded membranes for various applications such as zero mode wave guide structures for simultaneous electrical and optical measurements [6] and the fabrication of ion transistor structures [7–10] has been increasing. For these metal-containing structures, e-beam lithography and dry etching were commonly used to generate nanopores. However, these processes have limitations in forming nanopores with diameters smaller than 10 nm. Therefore, studies on the formation of nanopores in metal membranes using focused e-beams have been conducted. In this article, we analyzed the direct atomic displacement cross-section in detail, calculated $\upsigma_{\text{d}}$ as a function of the e-beam energy for various metals, then evaluated nanopore drilling as a function of atomic mass and displacement energy for selected metals, and finally performed nanopore perforation in various metal membranes (Ti, Cr, and Cu) using 200 kV TEM.

### Theoretical calculation of $\upsigma_{\text{d}}$

The equation for calculating $\upsigma_{\text{d}}$ is given by the Mckinley–Feshbach approximation [11] to the Mott and Massey expression [12] for high energy electrons as shown below [13]:

$$\upsigma_{\text{d}} = 4\pi a_{0}^{2} E_{R}^{2} Z^{2} \left( {\frac{{1 - \beta^{2} }}{{m^{2} c^{4} \beta^{4} }}} \right)\left[ {\frac{{E_{max} }}{{E_{d} }} + 2\pi \alpha \beta \sqrt {\frac{{E_{max} }}{{E_{d} }}} - \left( {\beta^{2} + \pi \alpha \beta } \right)\ln \left( {\frac{{E_{max} }}{{E_{d} }}} \right) - \left( {1 + 2\pi \alpha \beta } \right)} \right]$$

where ${\text{a}}_{0}$ is the Bohr radius ($5.29 \times 10^{ - 11} {\text{m}}$), ${\text{E}}_{\text{R}}$ is the Rydberg energy for hydrogen (${\text{E}}_{\text{R}} = \frac{{m_{0} e^{4} }}{{2\hbar^{2} }} = 13.606 \,eV$), $\upalpha = \frac{{{\text{Ze}}^{2} }}{\hbar c} = {\text{Z}}/137$, $\upbeta$ is a relativistic correction factor ($\upbeta = {\text{v}}/{\text{c}} = \sqrt {1 - \left( {1 + E/\left( {mc^{2} } \right)} \right)^{ - 2} }$), and Z is atomic number of the target atom. In addition, $E_{max}$ is the maximum transferred energy from the incident electron to the atom at rest ($E_{max} = 2E\left( {E + 2mc^{2} } \right)/Mc^{2}$), where m is the mass of an electron, and M is the mass of the target atom. Furthermore, ${\text{E}}_{\text{d}}$ is the atomic displacement energy required to move an atom from its original position while generating a defect.

Therefore, $\upsigma_{\text{d}}$ is a function of ${\text{E}}_{\text{d}} ,$ M, and E. To calculate $\upsigma_{\text{d}}$ of metals, ${\text{E}}_{\text{d}}$ should be determined first. The value of ${\text{E}}_{\text{d}}$ is related to the bonding energy because inter-atomic bonds should be broken to dislocate an atom from its original lattice point. Sublimation energy (${\text{E}}_{\text{sub}}$) (i.e., the cohesive energy or bond dissociation energy [14]) is the energy required to convert a substance from solid to gas, and can be calculated from thermodynamic data. Therefore, this value is strongly correlated with the melting point. The reported ${\text{E}}_{\text{d}}$ and calculated ${\text{E}}_{\text{sub}}$ of various metallic elements are shown in Table 1 [15]. For metallic elements, ${\text{E}}_{\text{d}}$ in the bulk is reported to be approximately ${\text{E}}_{\text{sub}} \times 4\sim 5$ [16]. The sputtering energy (${\text{E}}_{\text{s}}$), which represents ${\text{E}}_{\text{d}}$ of the atoms at the surface of a sample, is smaller than ${\text{E}}_{\text{d}}$ in the bulk because atoms at the surface are less tightly bond than those in the bulk, and therefore, ${\text{E}}_{\text{s}} \sim 1/2{\text{E}}_{\text{d}}$, usually [17, 18]. Table 1 shows the estimated range of ${\text{E}}_{\text{s}} \left( { 1. 5 {\text{E}}_{\text{sub}} < {\text{E}}_{\text{s}} < 2.5{\text{E}}_{\text{sub}} } \right)$.

**Table 1:** Calculations and literature survey of ${\text{E}}_{\text{d}} ,\;{\text{E}}_{\text{sub}} ,\;{\text{and}}\;{\text{E}}_{\text{s}}$ for various metals

Metal	Atomic number	Atomic weight	E_d_	E_sub_	E_s_	M.P. (°C)	Structure
Al	13	26.98	16	3.4	5.1–8.5	660	FCC^a^
Ti	22	47.87	15	4.9	7.4–12.3	1660	HCP^b^
V	23	50.94	29	5.3	8.0–13.3	1890	BCC^c^
Cr	24	52	22	4.1	6.2–10.3	1857	BCC
Fe	26	55.85	16	4.3	6.5–10.8	1535	BCC
Ni	28	58.69	22	4.5	6.8–11.3	1453	FCC
Co	27	58.93	23	4.4	6.6–11.0	1495	HCP
Cu	29	63.55	18	3.5	5.3–8.8	1083	FCC
Zn	30	65.39	16	1.4	2.1–3.5	420	HCP
Nb	41	92.91	24	7.5	11.3–18.8	2468	BCC
Mo	42	95.94	27	6.8	10.2–17.0	2617	BCC
Ag	47	107.87	28	2.9	4.4–7.3	962	FCC
Cd	48	112.41	20	1.2	1.8–3.0	321	HCP
Ta	73	180.95	33	8.1	12.2–20.3	2996	BCC
Pt	78	195.08	33	5.9	8.9–14.8	1772	FCC
Au	79	196.97	36	3.8	5.7–9.5	1064	FCC

^a^FCC face centered cubic

^b^HCP hexagonal close packed

^c^BCC body centered cubic

**Fig. 1:**
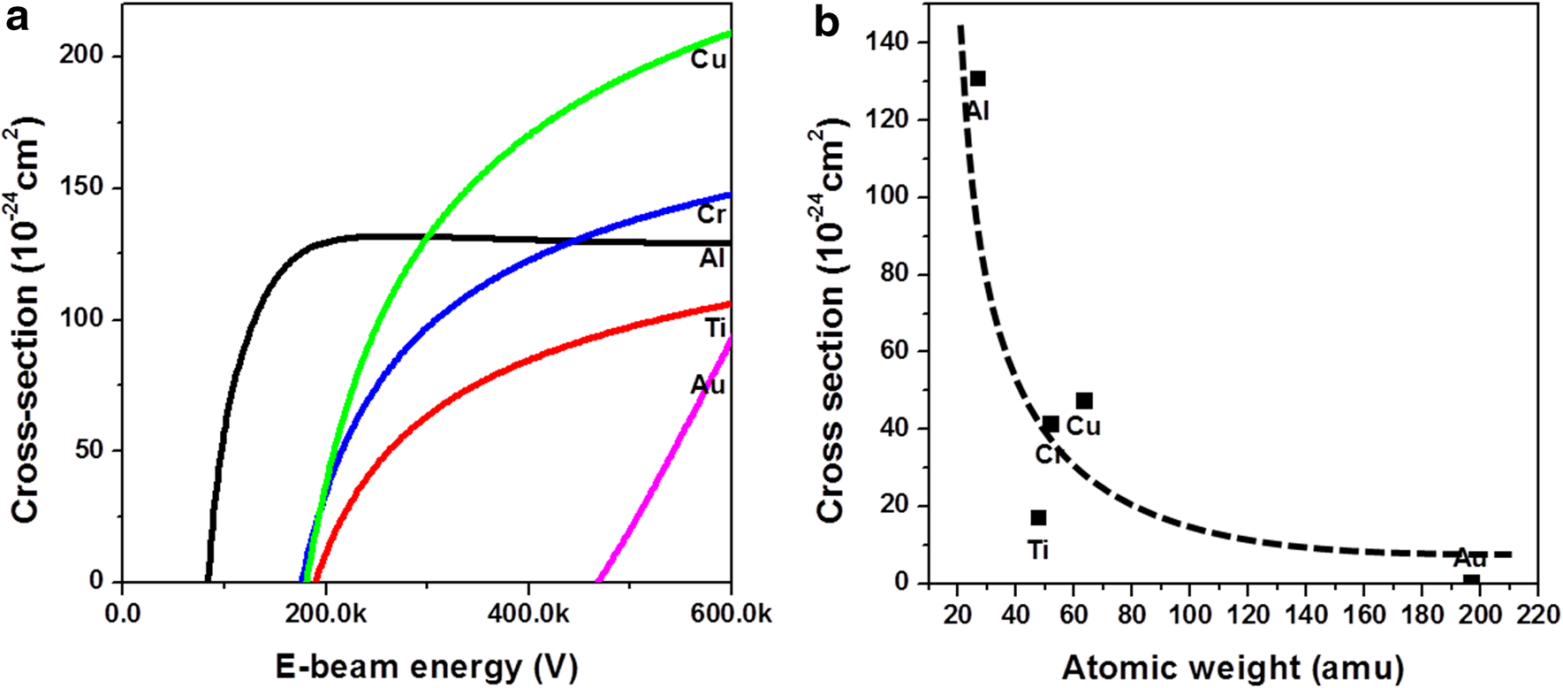
**a** Calculated $\upsigma_{\text{d}}$ as a function of E and **b** $\upsigma_{\text{d}}$ as a function of atomic weight for E = 200 kV for Al, Ti, Cr, Cu, and Au

We calculated $\upsigma_{\text{d}}$ of metals using the middle values of the estimated energy ranges of ${\text{E}}_{\text{s}}$ as ${\text{E}}_{\text{d}}$. From the calculated $\upsigma_{\text{d}}$ values, metals can be classified into three categories: (1) lightweight metals (Al) or low ${\text{E}}_{\text{sub}}$ metals (Zn and Cd), (2) heavy metals with high ${\text{E}}_{\text{sub}}$ (Ag, Au, and Pt), and (3) transition metals (Ti, Cr, Cu, Co, etc.). Figure 1a shows the calculated $\upsigma_{\text{d}}$ of Al, Ti, Cr, Cu, and Au as a function of E. Al, which is a light metal (27 amu) can be sputtered using e-beams with low energy, E < 85 kV, while Au, a heavier metal (79 amu) can be sputtered using e-beams with high energy, E > 450 kV. As can be seen in Fig. 1b, at 200 kV, $\upsigma_{\text{d}}$ starts decreasing with the increase in atomic mass. Generally, $\upsigma_{\text{d}}$ decreases with the increase in atomic mass [19]. The critical dependence of $\upsigma_{\text{d}}$ on the atomic mass of the target could explain the experimental results from previous reports, selective dissipation of light element such as oxygen and nitrogen in oxide and nitride [2, 20, 21] and decrease in milling rate with increasing the atomic mass of the metal [19]. While M_Ti_ (22 amu) < M_Cr_ (24 amu) < M_Cu_ (29 amu), the corresponding ${\text{E}}_{\text{sub}}$ values for these metals showed the inverse trend: ${\text{E}}_{\text{sub}} \left( {{\text{Ti}},{ 4}. 9 {\text{eV}}} \right) > {\text{E}}_{\text{sub}} \left( {{\text{Cr}},{ 4}. 1 {\text{eV}}} \right) > {\text{E}}_{\text{sub}} \left( {{\text{Cu}},{ 3}. 5 {\text{eV}}} \right)$. As can be seen in Fig. 1b, the calculated $\upsigma_{\text{d}}$ values of Ti, Cr, and Cu at 200 kV follow the same trend as ${\text{E}}_{\text{sub}}$.

## Experimental

We evaporated 30 nm thick Ti, Cr, Cu, and Au films on ultra-thin carbon film in Cu grids. For drilling and observing the nanopores, the samples were loaded in a TEM instrument with 200 kV field emission guns (JEM-2010F). The nanopores were drilled using the focused 200 kV e-beam of the TEM instrument by varying the size of the aperture of the condenser lens (CL). The aperture sizes of the CL used for our study were 150, 100, and 70 μm and the total e-beam current values 7, 3, and 1.6 nA. The total e-beam current was measured using the viewing screen of a Keithley 6430 source meter [22]. Therefore, we were able to precisely calculate the electron flux (e/nm^2^ s) by dividing the electron current (C/s) by the exposure area (nm^2^). The exposure area was determined from the size and magnification of the e-beam on the viewing screen.

## Results and discussion

**Fig. 2:**
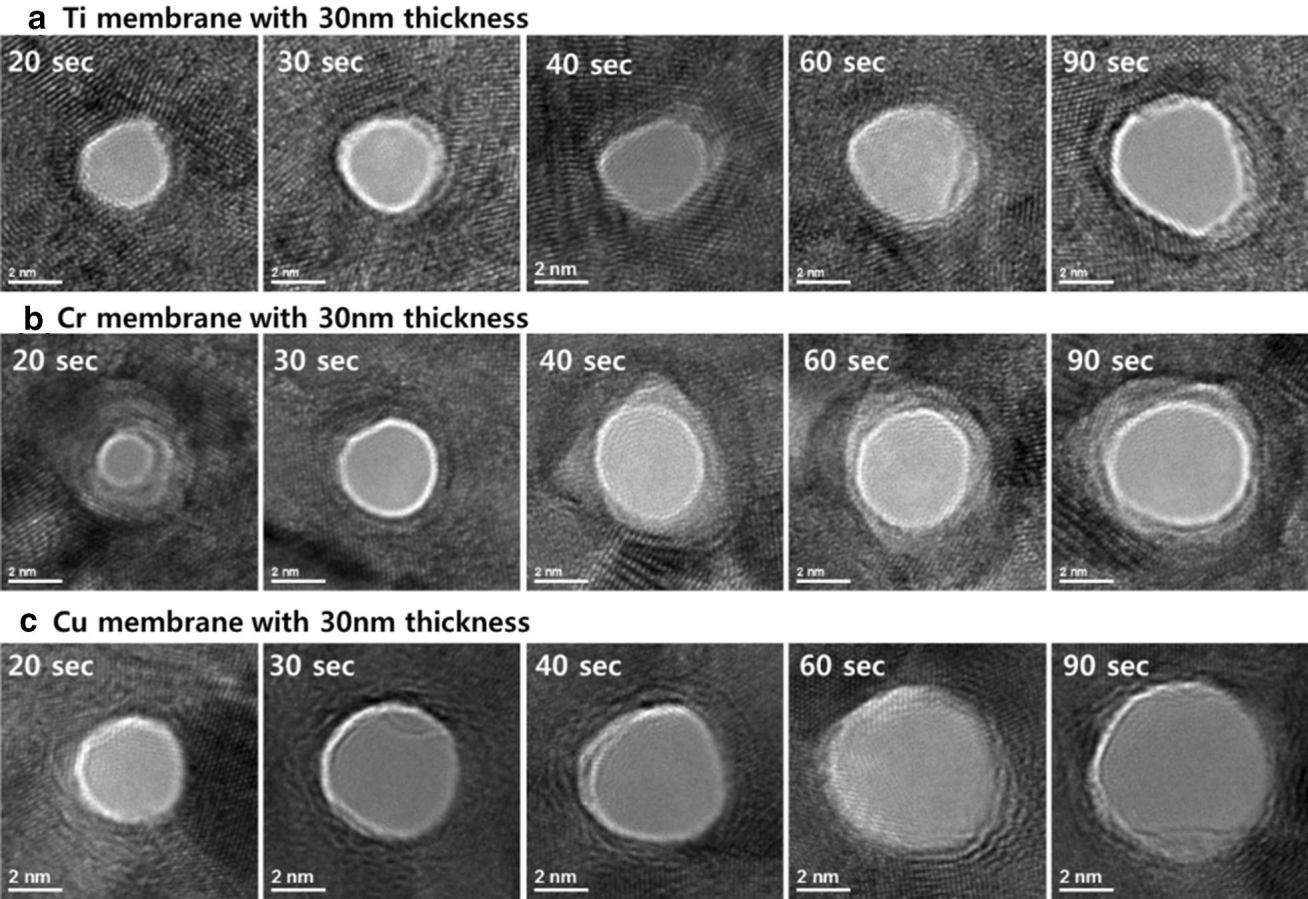
Nanopore evolution for 30 nm thick **a** Ti, **b** Cr, and **c** Cu membranes with e-beam exposure time, at 200 kV and 7 nA

Nanopores were drilled in various 30 nm thick metal membranes (Ti, Cr, Cu, and Au) using a 200 kV focused e-beam and different probe sizes by changing the aperture of the CL. Figure 2a–c shows the TEM images of the evolution of nanopores in the Ti, Cr, and Cu membranes, respectively, as the e-beam exposure time increased from 20 to 90 s at an e-beam current of 7 nA. The 30 nm thick Au membrane could not be drilled using the 200 kV e-beam; this E value coincided with a zero $\upsigma_{\text{d}}$ calculated value for Au. After the initial 20 s of e-beam exposure, the perforation occurred in a stable manner, and the minimum diameters of the nanopores were similar and approximately 2 nm each. The diameters of the nanopores gradually increased as the e-beam exposure time increased. The increase in e-beam exposure time brought changes in the diameter of the nanopores. After 90 s of exposure, the diameters of the nanopores for the Ti, Cr, and Cu membranes were 4.5, 5, and 6 nm, respectively.

**Fig. 3:**
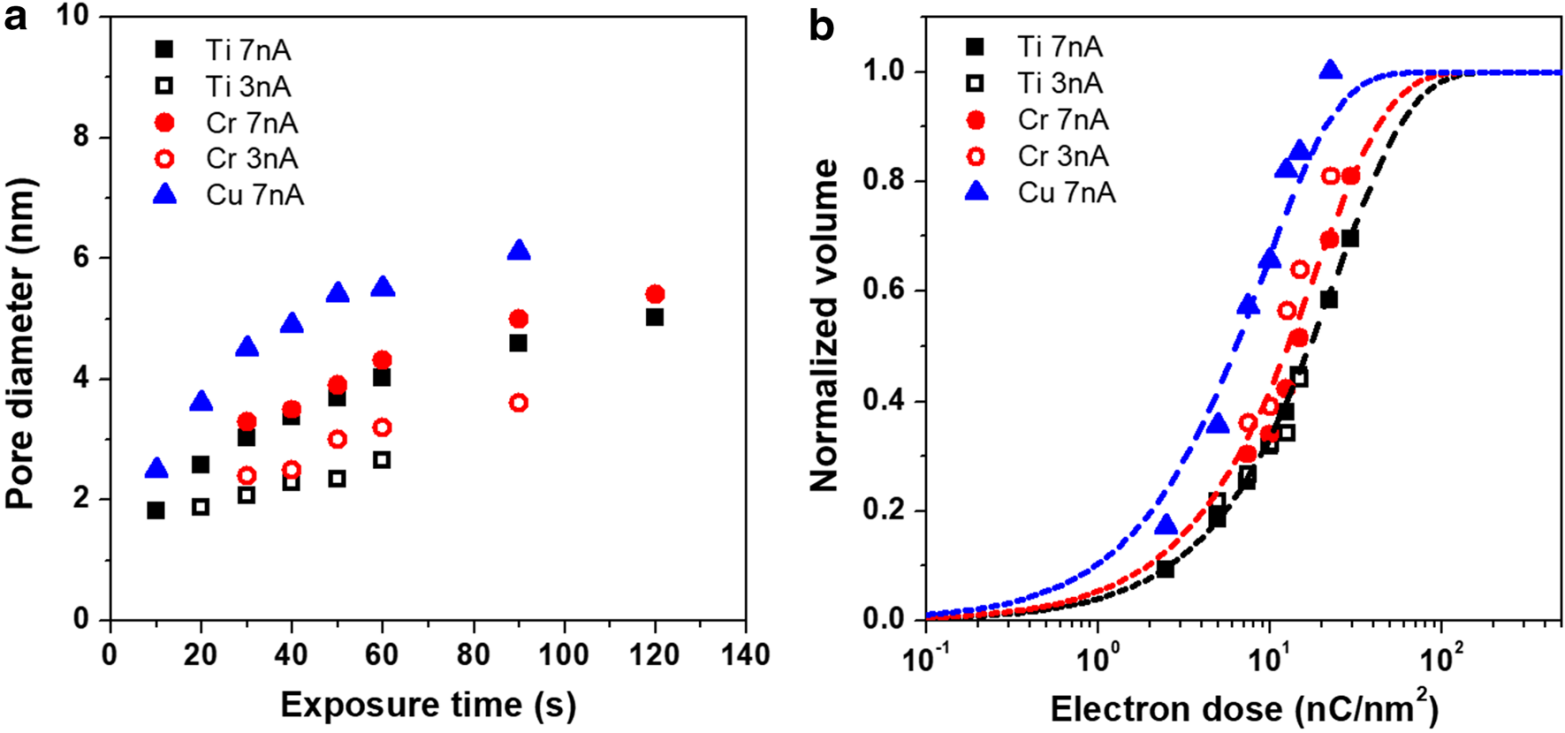
**a** Pore diameters of Ti, Cr, and Cu membranes as a function of e-beam exposure time and **b** contrast curve of nanopore drilling

Figure 3a shows the resulting pore diameters as a function of the e-beam exposure time for various metal membranes for different e-beam current values. The square, circle, and triangle correspond to the Ti, Cr, and Cu membranes, and the solid and open dots correspond to the 7 and 3 nA e-beam currents, respectively. For all experiments, the diameters increased with increasing the exposure time and eventually peaked. The largest diameters were 3 and 6 nm at 3 and 7 nA, respectively, which corresponded to the increase in the e-beam probe size by ~ 2 and ~ 3 nm, respectively. Reaching the peak pore diameter is a major advantage of nanopore perforation using focused e-beams. The increase in nanopore diameters was similar for the Ti and Cr membranes. The increase in pore diameter for the Cu membrane occurred faster than for other membranes, although Cu was the heaviest of the three analyzed metals. Since the e-beam current and probe size simultaneously changed as the e-beam current through the aperture of the CL in the TEM instrument changed, we introduced the characteristic contrast curve plotting the normalized drilling volume as a function of the electron dose (nA/cm^2^) to define the drilling amount as the electron dose. Therefore, we changed the plot of the diameter vs. exposure time to that of the normalized drilling volume vs. electron dose, as shown in Fig. 3b.

The e-beam exposure time can be converted into electron dose (nC/nm^2^), representing the number of electrons impinging on the membrane per unit area. To obtain the normalized volume, the drilled volume was calculated first using the formula: $\left[ {\uppi \times \left( {{\text{diameter}}/2} \right)^{2} \times {\text{thickness }}} \right]$, assuming a cylindrical shape and, then, was normalized by the saturated drilled volume. Figure 3b shows the contrast curve of nanopore drilling for different metal membranes. It was revealed that the same dose of electrons was needed for the same drilling volume and the same metal membrane. In addition, it is worth noting that the contrast curve shifted toward higher electron doses in order from Cu to Cr and Ti.

Under the assumption that atoms are removed from the specimen by the interaction with the e-beam, the reacted volume can be expressed by the simple reaction kinetics for exponential decay [21, 23]:

$$\frac{{{\text{N}}_{\text{d}} }}{{{\text{N}}_{ 0} }} = 1 - \exp \left[ {\left( { - \sigma Jt} \right)^{n} } \right]$$

where ${\text{N}}_{\text{d}}$ is the number of drilled atoms, ${\text{N}}_{0}$ is the initial number of atoms, $\upsigma$ is the cross section (m^2^), J is the electron current density (e/s m^2^), t is the exposure time (s) and n is the kinetic order. Here, ${\text{N}}_{\text{d}} /{\text{N}}_{0}$ is the normalized volume. We performed the fitting of Fig. 3b using this equation. The slope of the contrast curve represents the reaction order for the pure metal sample, and is well fitted with the first order reaction (n = 1), which is different for the Si_3_N_4_ [5] and TiN membranes [24] (n = 2). It has been reported that light element of oxides and nitrides selectively dissipate after their initial exposure to the e-beam [21]. Consequently, it is believed that the higher kinetic orders of oxide and nitride membranes are caused by the promotion of overall atomic dissipation due to light element sputtering.

**Fig. 4:**
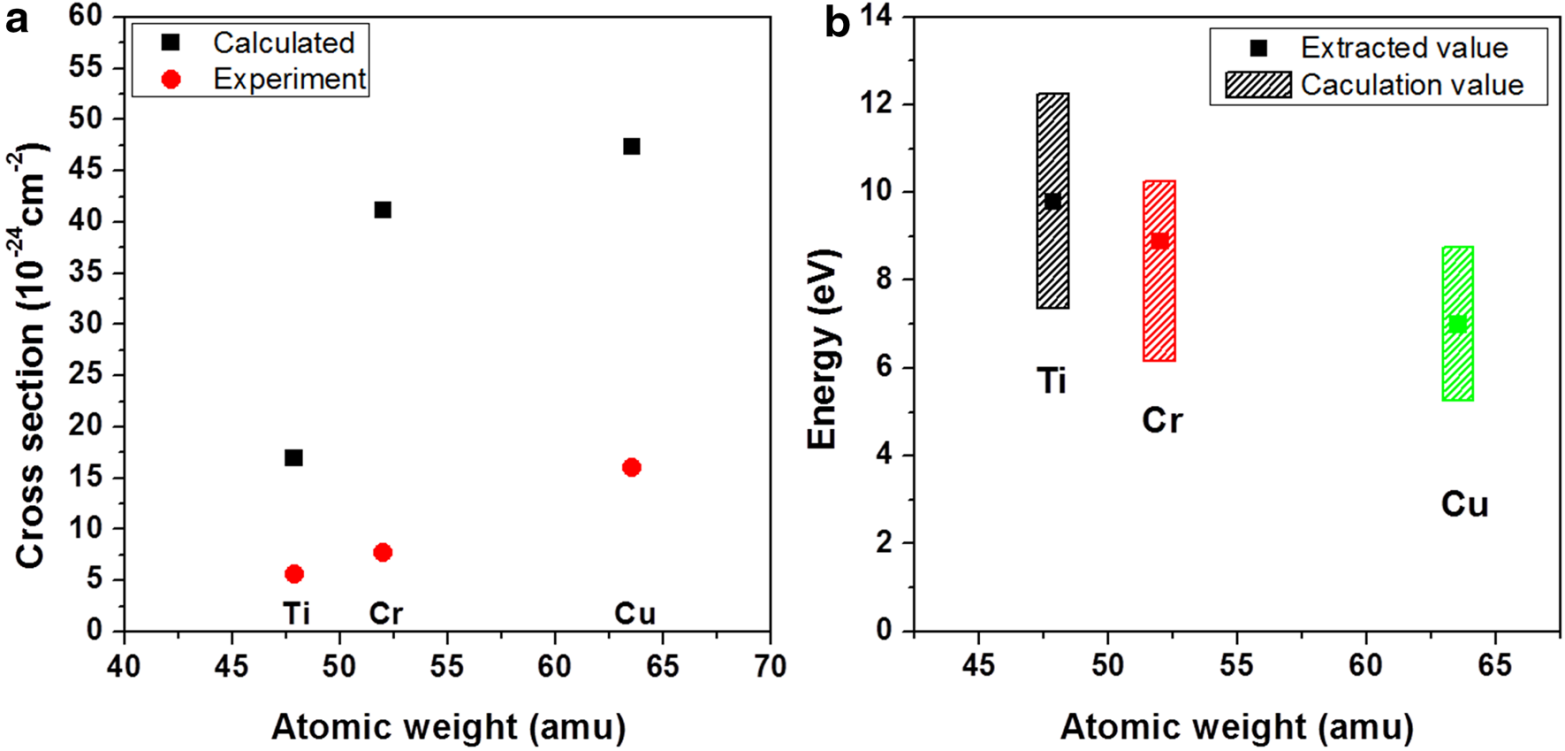
Calculated vs. experimental values of **a** displacement cross-section and **b** displacement energy

From the fitting of the contrast curve, we were able to obtain the atomic displacement cross-sections (sputtering cross-sections) and sputtering energies for perforation. Figure 4a and b shows the theoretical calculations and experimental values of sputtering cross-sections and energies, respectively. The experimentally obtained cross-section values were closer to the elastic scattering cross section value (~ 10^−24^ cm^2^) and the tendency of the experimental values to decrease as the atomic weight increased was in agreement with that of the calculated values based on the knock-on damage in Fig. 1. This dependency of the contrast curve on the membrane material demonstrated that nanopore drilling was mainly controlled by the direct atomic displacement. The sputtering energies of Ti, Cr, and Cu determined from the experimental sputtering cross-sections in Fig. 4a were approximately 10, 9, and 7 eV, respectively, which were in the range of the ${\text{E}}_{\text{s}}$ values reported in Table 1 (Fig. 4b). Therefore, nanopore evolution kinetics when using high energy e-beams was determined by the bonding energy as well as the atomic mass of the target atom. When the membrane material is the same, the atomic mass does not change, but the bonding status can vary due to the fabrication method. Therefore, the sputtering energy required to remove the surface atoms is influenced by the bonding state of the surface atoms, which would influence the scattering cross-section.

## Summary

We first summarized the two interaction mechanisms between the fast electrons and atoms at rest, viz. the elastic scattering accompanying the direct atomic displacement caused by the kinetic energy transfer and the inelastic scattering involving the ionization or excitation caused by the electron–electron collisions. We concluded that nanopore drilling using focused e-beams was governed by the direct atomic displacement or sputtering. We calculated the direct atomic displacement cross-sections of several metals. Al, Ti, Cr, Cu, and Au were selected to investigate the effect of the displacement energy and atomic mass on nanopore drilling. As the atomic mass increased, the effect of the elastic scattering decreased, overall. We were unable to drill nanopores in the 30 nm thick Au membrane using a 200 kV e-beam. We investigated the evolution of nanopores with the e-beam exposure time and obtained characteristic contrast curves for the Ti, Cr, and Cu membranes. While M_Ti_ < M_Cr_ < M_Cu_, the corresponding ${\text{E}}_{\text{sub}}$ values for these metals showed an opposite trend. The experimental cross-sections obtained from the contrast curves were good matches for the calculated cross-sections based on elastic scattering. The sputtering energies of Ti, Cr, and Cu determined using the experimental sputtering cross-section were approximately 10, 9, and 7 eV, respectively, and were within the normally accepted sputtering energy range calculated using the ${\text{E}}_{\text{sub}}$ values.
